# Cancer of the Liver

**Published:** 1870-09

**Authors:** C. W. Earle


### CHICAGO MEDICAL EXAMINER.

N. S. DAVIS, M.D., Editor.

VOL. XI. SEPTEMBER, 1870. NO. 9.

### Original Contributions.

ARTICLE XXIII.

### CANCER OF THE LIVER.
### WITH CLINICAL LECTURE BY PROF. N. S. DAVIS.

By C. W. EARLE, M.D.

James Stableton, white, Irish nativity, blacksmith, aged about 31.

History.—This man gave a family history from which we were unable to determine that he was liable to any hereditary complaint. He has always enjoyed the best of health until within the last fourteen months; having been from his youth an active laborer. Some six months previous to his admission to the Hospital, he received a blow on his head, which caused him some pain at the time, and from the effects of which he is inclined to attribute all his present symptoms.

He complains of great pain in the region of the stomach. He describes it as *burning*. The pain in his left side is paroxysmal, and frequently extends up toward the shoulder and around on the left side, toward the spine. He also has a very severe headache, sweats very profusely, poor appetite. He was also troubled with vomiting. Immediately upon admission, he was subjected to a close examination, but neither percussion, auscultation, or palpation gave any clue to his disease. The patient was placed on palliative treatment, and all his symptoms closely observed. To be sure, the continued pain in his stomach, still radiating, combined with a fetid breath and a decided cachectic appearance, which I should have mentioned before, pointed strongly to malignant disease of that organ. Yet, there was the absence of some symptoms that we always expect in cancer of the stomach, and so the diagnosis was withheld. About this time, some three weeks after his admission to Hospital, a change in the location of his pain, and an examination as to its cause revealed the precise condition of our patient.

The pain in the vicinity of the stomach was almost entirely relieved, and his suffering was now referred to his right side. By flexing his legs and relaxing the abdominal muscles, an enlarged and hardened liver could easily be detected.

DIAGNOSIS.— Cancer of the liver.

The clinical lecture delivered by Prof. N. S. Davis at the patient's bedside, gave more practical instruction in the case than can be found elsewhere, and I therefore feel at liberty to transcribe from my note-book his remarks:

"GENTLEMEN:— The general history of the case now before you has been given by the house physician, and I need not repeat. The symptoms at first were so referrable to his stomach, that I regard it important that you should be made familiar with diseases of this organ, characterized by pain, tenderness, etc., and the differential diagnosis between them.

"In Chronic Gastritis, you would generally find that food increased the pain. There would be occasional vomiting and constipation, emaciation not very marked, and no cachectic appearance. We have, too, a curable disease, and, of course, would not expect to find any tumor.

"In Gastric Ulcer, we have a localized pain — paroxysmal, but not lancinating, generally, hemorrhage from the stomach, debility, etc., etc. The prognosis is not as favorable as in Chronic Gastritis, as it may go on to perforation, yet many cases get well in time, yielding to proper treatment.

"In Cancer of the Stomach, we have a radiating pain, severe and lancinating. Vomiting is a very frequent symptom, and is described by the authors as always being present. The bowels are constipated, and we notice a constant wasting of flesh, with cachexiæ. A tumor may generally be detected by close examination, and I think some swelling or change in the outline of the organ may be observed. This patient. you will notice, had certainly some of the symptoms of cancer of the stomach. There was the severe and lancinating pain with the cachectic appearance, the debility and emaciation. But the pain from which he is now suffering is in the vicinity of the liver, and by placing the patient on his back and flexing his limbs, you can distinctly feel the organ enlarged. You will notice that this enlarged and hardened mass emerges from under, and extends to a point two inches below the ribs, to the left it reaches to the ensiform appendix, to the right to the notch that separates the right and left lobes. You will most always be able to detect by percussion, the boundary of the liver in its position under the ribs—one-half inch above the point of the ensiform appendix, directly across toward the right, marking the superior normal border of the organ. This enlargement is in the same position that we would find an enlarged pylorus, but the widest part is above, which would not be true with an enlarged pylorus. The fact that it is not only broadest above but continues upward under the ribs and to the right, occupying the right hypochondriac space, shows that it actually includes the liver. We may also have an encephaloid growth of a kidney, which must be diagnosed from the diseased condition now before us. Always trace its *commencement*, and you will find little trouble as a general thing, in distinguishing the organ effected.

"In Disease of the Kidney, if the organ was enlarged, the free margin would point toward the mesial line, and the analysis of the urine would be of great assistance. An ovarian tumor sometimes fills the whole abdominal cavity; but it rises from the lower part of the abdomen, which, with other symptoms familiar to you, would enable you to make out a diagnosis. Any of the mesenteric glands may take on an enlargement, but by observing carefully the directions which I have given to trace them to their origin, and note all other symptoms, you will experience but little difficulty in arriving at the correct condition.

"Now, as to the nature of the tumor before us. There is no stoppage of ducts, as we have no jaundice. This peculiar color of the skin which you have all noticed is not that of a jaundiced condition. It is rather from a want of red corpuscles in the blood, and not from the want of proper excretion of bile matter. This cannot be a distended gall bladder, as the tumor is too large; and if such was the case, we could trace its shape. This is certainly an enlarged liver. What is its character? Is it simple inflammation, fatty degeneration, or cancer? If from the first, either acute or chronic, he would have noticed more or less febrile excitement, with nausea and vomiting, derangement of the bowels, etc., etc. If this enlarged organ was from fatty degeneration, the patient would never have experienced the acute, lancinating pain which he has been obliged to suffer. With this affection there would have been a more progressive loss of strength, with less emaciation, and less active disturbance of the system. It would have been usual in a short time to have noticed some dropsical condition in fatty degeneration, while in cancer it is generally wanting. Its persistence, the hardness, the general cachexia, all indicate cancer. The prognosis is extremely unfavorable. No curative treatment has as yet been discovered; yet we may do much to alleviate the sufferings of our patient, if not wholly retard the progress of the disease. An exudation or induration from inflammatory action can usually be controlled by remedies, but a new growth, with enough vitality to produce cell formation, is seldom controlled by remedial agents.

"Treatment.—Pay particular attention to the diet. Milk and farinacious articles are the best. Meat should be used only sparingly. For an internal remedy, I have realized the most benefit from some of the arsenical preparations with conium. The following is a good formula:

"℞ Arsenite Soda,------------------------------grs. iv.
Ext. Conium,--------------------------------ʒjss.

"Make a mass, and divide into sixty pills.

"Sig. Take one three or four times a day.

"Anodynes sufficient to relieve pain should be given, selecting those that do not constipate, if possible. Among the best are Fluid Ext. Hops, and Lettuce. By such treatment, you can do much to palliate the sufferings of the patient, and delay somewhat the fatal result.

"The patient which was the subject of the foregoing clinic, continued to fail until May 17th, when he died, apparently from copious hemorrhage from the bowels. His entire sickness was about eight months duration.

"AUTOPSY.—Twenty-four hours after death, considerable emaciation was noticed—integument a peculiar yellow color—pupils normal—rigor mortis well marked.

"SECTIO-CADAVERIS.—1. Head not opened. 2. Lungs and Heart normal. 3. Stomach inflated with gas, which, being collapsed, revealed a large abscess, probably originating in the glands near the cardiac extremity of the stomach. A great amount of pus and disintegrated matter was in the cavity, but no hardened edges, or any indication of its being of malignant origin. Liver very much enlarged. Weight 7¾ pounds. The left lobe extended to just across the mesial line, and was hardened and mottled. The right lobe was *very much* enlarged, filling the whole hypochondriac space, and crowding the diaphragm upward. Two large, rounded nodules, retracted in the center, occupied a great part of this lobe. Cutting through this, it had every appearance of being cancerous. The remaining viscera appeared perfectly healthy, excepting some of the mesenteric glands. At least, in one locality we found, upon opening a gland, what appeared to be the peculiar cancroid substance.

"REMARKS.—This case is very interesting, as we were able to prove our diagnosis, and also learn the cause of our perplexities and errors before we made the diagnosis. It is highly probable that the disease of the liver existed before his admission to the Hospital, but had not caused sufficient enlargement to be readily detected. The abscess connected with the stomach also existed, but it was more inflammatory, and caused all the symptoms of gastritis."

NOTE.—The reader will remember that in the August number of the *Examiner*, an Editorial statement was made in regard to this case. A specimen of the carncerous tissue was examined under the microscope, but none of the cells supposed to be characteristic of cancer were found.

This brings us to the much discussed question as to the value of the microscope in the diagnosis of cancer, which, in simply recording the case, is not our province to discuss.

